# X-ray phase-contrast imaging with engineered porous materials over 50 keV 

**DOI:** 10.1107/S1600577518005623

**Published:** 2018-06-12

**Authors:** Hongchang Wang, Biao Cai, Matthew James Pankhurst, Tunhe Zhou, Yogesh Kashyap, Robert Atwood, Nolwenn Le Gall, Peter Lee, Michael Drakopoulos, Kawal Sawhney

**Affiliations:** a Diamond Light Source, Harwell Science & Innovation Campus, Didcot OX11 0DE, UK; b School of Metallurgy and Materials, University of Birmingham, Birmingham B15 2TT, UK; c School of Materials, University of Manchester, Manchester M13 9PL, UK; d Research Complex at Harwell, Rutherford Appleton Laboratory, Harwell, Oxfordshire OX11 0FA, UK; e School of Earth and Environment, University of Leeds, Leeds LS29 9ET, UK; f Instituto Technológico y de Energías Renovables (ITER), 38900 Granadilla de Abona, Tenerife, Canary Islands, Spain; g Instituto Volcanológico de Canaries (INVOLCAN), 38400 Puerto de la Cruz, Tenerife, Canary Islands, Spain; h Technical Physics Division, Bhabha Atomic Research Centre, Mumbai 400085, India

**Keywords:** X-ray phase imaging, speckle technique, hard X-rays, random attenuation masks, high-energy regions, porous materials

## Abstract

X-ray phase-contrast imaging can substantially enhance image contrast for weakly absorbing samples. The fabrication of dedicated optics remains a major barrier, especially in high-energy regions (*i.e.* over 50 keV). Here, the authors perform X-ray phase-contrast imaging by using engineered porous materials as random absorption masks, which provides an alternative solution to extend X-ray phase-contrast imaging into previously challenging higher energy regions. The authors have measured various samples to demonstrate the feasibility of the proposed engineering materials. This technique could potentially be useful for studying samples across a wide range of applications and disciplines.

## Abstract 2

Engineered porous materials are proposed as random absorption masks for X-ray phase-contrast imaging in high-energy regions (*i.e.* over 50 keV).

## 1. Introduction   

High-energy X-ray imaging permits the study of large and highly attenuating samples for advanced research in geo-sciences, biomedical engineering, civil engineering and material science (Cnudde & Boone, 2013 ▸; Baars *et al.*, 2013 ▸; Zhang *et al.*, 2013 ▸; Cai *et al.*, 2015 ▸; Karagadde *et al.*, 2015 ▸). Over the last few decades, numerous exciting studies have been carried out with high-energy X-rays. These have been enabled by the rapid development of brilliant synchrotron radiation sources, advanced X-ray optics and highly efficient imaging detectors. For example, tomographic absorption imaging of rocks has grown in popularity in research and applied settings across the geosciences (Reyes-Dávila *et al.*, 2016 ▸; Jerram & Higgins, 2007 ▸).

However, it should be noted that advances in high-energy X-ray imaging have come at an inherent cost to absorption contrast because of the reduced absorption cross-section for most materials with increasing X-ray energy. Inevitably, the absorption images cannot be easily segmented between some key textural and chemical components. In contrast, X-ray phase-imaging techniques can enhance image contrast and retrieve complementary information to absorption-contrast imaging (Willner *et al.*, 2013 ▸; Olivo *et al.*, 2012 ▸; Nesch *et al.*, 2009 ▸; Donath *et al.*, 2009 ▸; Vittoria *et al.*, 2015 ▸). For example, the edge-illumination technique was demonstrated to achieve intense phase signals at 85 keV (Olivo *et al.*, 2012 ▸; Endrizzi *et al.*, 2014 ▸), and grating interferometery was used to quantify X-ray refractive-indices above 100 keV (Ruiz-Yaniz *et al.*, 2015 ▸). High-energy X-ray phase-contrast imaging provides an opportunity to better distinguish between these components, enabling observation and measurement.

Here, we report an advance in phase-contrast capability using the speckle technique in the high-energy X-ray regime. To overcome the use of sophisticated fabrication steps, we first explored the use of a metal alloy as a random absorption mask (RAM) and used rocks and biological material as test cases. The encouraging results led us to conduct further tests to see how this technique might be optimized for different purposes. It was found that different RAMs can improve speckle visibility and image quality. We believe that this opens the door to achieve high-quality X-ray phase-contrast images in high-energy regimes and reveal additional information from samples that would otherwise be intractable.

## 2. The speckle technique   

The speckle-based technique is a recently developed X-ray phase-imaging method that shows great potential to overcome the fabrication issue (Berujon, Ziegler *et al.*, 2012 ▸; Morgan *et al.*, 2012 ▸; Wang, Berujon *et al.*, 2015 ▸). It provides quantitative and multimodal images (absorption, phase and dark-field). It also permits the study of materials with either high speed or high spatial resolution by using single-image tracking mode or multi-image scanning mode (Berujon *et al.*, 2013 ▸; Berujon, Wang *et al.*, 2012 ▸; Wang, Kashyap *et al.*, 2016*a* ▸,*b* ▸).

**Figure 1:**
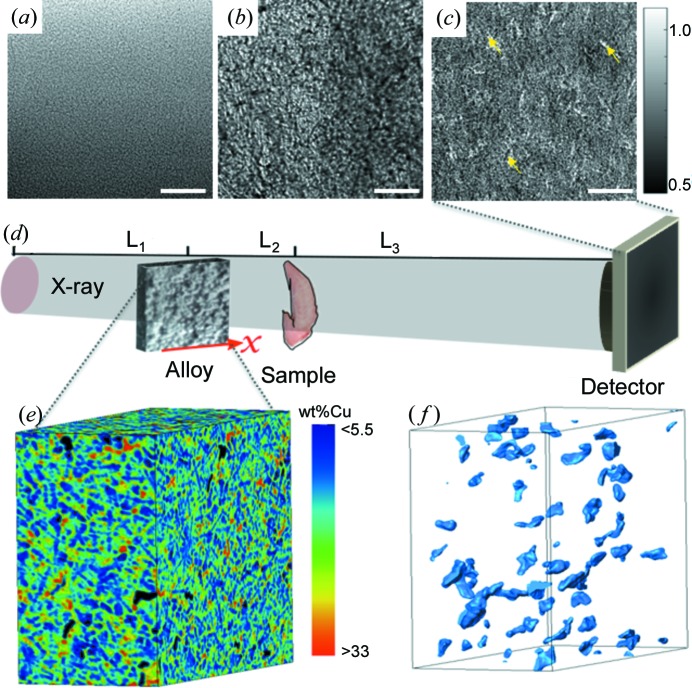
Methodology schematic and detailed structure of a RAM highlighting useful qualities. X-ray projection image of (*a*) abrasive paper, (*b*) steel wool and (*c*) Al–Cu alloy; the scale bars of these images are 1.0 mm long. Some pores are marked with yellow arrows in the speckle image (*c*). (*d*) Schematic representation of the experiment setup (not to scale). For each projection a stack of speckle images was recorded using an X-ray detector by scanning alloy transversely (along the *x* direction) to the X-ray beam. (*e*) Volume rendering of an X-ray tomographic image of the Al–Cu alloy (the black colour represents gas porosity), (*f*) porosity in the alloy. Here, the size of the tomographic image is 1695 µm × 918 µm × 2124 µm.

In the high-energy region, the success of this technique relies on the choice of a RAM. It is desirable that the materials used as the RAM should fulfill the following criteria: (i) have a random internal structure at a fine scale yet uniform spatial distribution at a broad scale, (ii) be able to generate the high-contrast random pattern, (iii) a wide range of structure sizes and (iv) be easily manufactured. Although the use of steel wool as RAM significantly improves the absorption contrast (Wang, Kashyap, Cai *et al.*, 2016 ▸), steel wool has a limited range of fiber thicknesses, which means it is difficult to find the right size of fiber to match the pixel size of the X-ray imaging system. Compared with coarse abrasive paper (P800), see Fig. 1(*a*) ▸, the average speckle size generated from finest steel wool is about three times larger (∼90 µm), see Fig. 1(*b*) ▸. In addition, the generation of a uniformly distributed random speckle pattern can be quite involved, and such nonuniformity can also be observed from the speckle image in Fig. 1(*b*) ▸. In this study, Al–Cu alloy, Mg–Zn alloy, limestone and mortar were chosen to demonstrate the feasibility of engineering and building materials used as RAMs.

## 3. Proof of concept   

To begin with, a hypo-eutectic Al-15 wt% Cu alloy (Cai *et al.*, 2016 ▸) was used as a RAM. The alloy was manufactured by a conventional casting method (melted at around 700°C and poured into a ceramic mold, then passively cooled to ambient). The Al–Cu alloy was firstly measured with a Nikon XT H 225 computed tomography system to inspect the internal structure. The volume rendering of an X-ray tomographic image of the Al–Cu alloy is shown in Fig. 1(*e*) ▸. The microstructure of the as-cast alloy contains three regions: large α-Al dendrites with composition close to Al-5 wt% Cu, fine Cu-enriched inter-dendritic region (eutectics, average composition close to Al-33 wt% Cu) and shrinkage pores (Cai *et al.*, 2014 ▸) formed in the casting process.

As shown in Fig. 1(*f*) ▸, the porosity is randomly distributed inside the Al–Cu alloy. The order of magnitude difference in Cu concentration in the solid regions and the existence of the porosity results in considerable X-ray absorption-contrast in the hard X-ray region. The complex dendritic patterns and the fine eutectic region, together with the gas pores, form uniform speckles at the length scale (a few µm to tens of µm) appropriate to be used as a RAM for our current X-ray imaging setup. Fig. 1(*c*) ▸ shows the generated speckle image at 53 keV with the proposed engineering alloy. The average speckle size is about 40 µm, and the visibility (ratio between standard deviation and mean) of the speckle is around 6% at 53 keV.

The first experiment was conducted by using a piece of Al–Cu alloy (thickness = 12 mm) on the I12 beamline at Diamond Light Source, UK (Drakopoulos *et al.*, 2015 ▸). Fig. 1(*d*) ▸ shows the experimental setup consisting of an Al–Cu alloy RAM, a sample and an X-ray area detector. The photon energy of the incident X-ray was set to 53 keV by a double Laue crystal monochromator. The Al–Cu alloy was mounted on a linear stage, which was located at a distance of 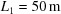 from the source. The sample was placed 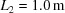 downstream of the Al–Cu alloy. The distance between the sample and the detector was set to 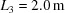. The speckle pattern was recorded by an X-ray imaging camera with a field of view of 20 mm × 17 mm and an effective pixel size of 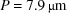.

For each sample, a stack of 50 speckle images was firstly taken without the sample present by scanning the Al–Cu alloy along a horizontal direction with a linear motor stage. The scanning step size was μ = 5 µm, and the exposure time for each image was 50 ms. Another stack of speckle images was then collected with the sample inserted into the beam. As part of the one-dimensional speckle scanning technique (Wang, Kashyap *et al.*, 2016*b* ▸), a speckle pattern for each pixel is generated by combining the nearby pixels with the series of data along the scanning direction. A cross-correlation algorithm is then applied between the reference speckle image and the corresponding sample speckle image. The maximum of the cross-correlation coefficient ξ can be precisely located with a sub-pixel registration algorithm (Bing *et al.*, 2006 ▸). The speckle displacement induced by the sample is related to the coordinate (ξ^*x*^, ξ^*y*^) of the maximum of the cross-correlation coefficient. For vertical scanning mode, the wavefront gradient (α^*x*^, α^*y*^) can be calculated from the speckle displacement by following a basic geometric relationship, 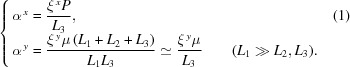
Once the wavefront gradients are derived, the phase shift induced by the sample can then be generated by applying two-dimensional integration from the retrieved transverse wavefront gradients (Kottler *et al.*, 2007 ▸).

To demonstrate the proposed RAM for advanced geological studies we investigated two representative volcanic rocks: picrite (from Iceland) and rhyolite (from New Zealand). The picrite (high Mg and Fe, low Si) is composed of large olivine crystals, and Cr-spinel occurs within olivine crystals, and along with Fe–Ti oxides are the most abundant accessory phase in the groundmass. In contrast, the rhyolite (low Mg and Fe, high Si) is composed of variable-sized pyroxene, plagioclase and volumetrically minor oxide phases set within a highly vesiculated glassy matrix. Parallel cross-sectional slices through cores with 14 mm diameter were made using a bench drill and Petrothin grinding apparatus. The thickness of the picrite and rhyolite sample was 530 µm and 780 µm, respectively.

**Figure 2:**
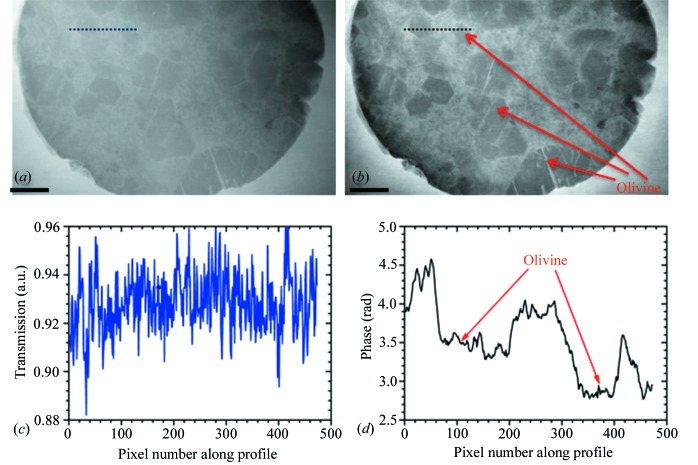
Hard X-ray speckle technique applied to a sample of picrite (very low absorption contrast). (*a*) Transmission and (*b*) phase image demonstrating improved definition of olivine crystal, and surrounding matrix. The scale bar at the bottom of the image is 2.0 mm long. (*c*, *d*) Line profiles of the image at the positions indicated by the thin lines from (*a*) and (*b*).

As shown in Fig. 2(*a*) ▸, the picrite sample exhibits poor absorption contrast between the olivine and the host groundmass, and it is thus difficult to apply digital segmentation. As expected, the image contrast has been significantly improved in the phase image (Fig. 2*b*) ▸. The olivine can be clearly identified from the host groundmass, which facilitates measurement. Line profiles for the absorption and phase images are highlighted in Figs. 2(*c*) ▸ and 2(*d*) ▸. Here, high spatial frequency features in the transmission image are due to the edge-enhancement effect. The line profiles in Fig. 2(*d*) ▸ clearly show the position of the olivine in the picrite sample. Further work is underway to retrieve the internal structures from X-ray phase tomography with the proposed RAM.

**Figure 3:**
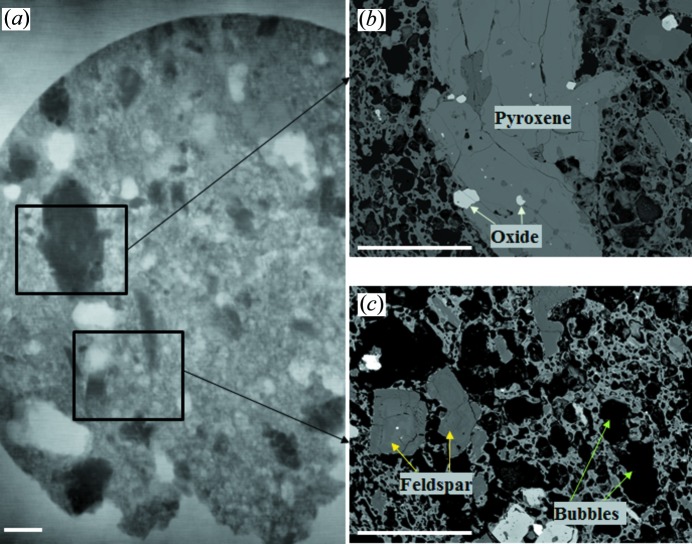
Comparison between imaging techniques using a rhyolite sample. (*a*) Phase-contrast image and (*b*, *c*) backscattered electron images. White scale bars are 1.0 mm.

Following the same procedure, the phase-contrast image of the rhyolite sample is shown in Fig. 3(*a*) ▸. Here, the bubbles (white), groundmass glass (light grey), feldspar (medium grey) and pyroxene (dark grey) can be clearly identified from the phase image. To verify these interpretations, the samples were set in ep­oxy resin and polished for *in situ* routine spot chemical analysis and conventional backscatter electron (surface) imaging after the X-ray imaging experiment. Backscattered electron images (Figs. 3*b*, 3*c*) ▸ were taken by using a Jeol 8230 Electron Probe Microanalyser at an energy of 20 kV. The greyscale backscattered electron images record signal intensity using a raster scan. Although some surface material was lost due to the polishing process, backscattered electron images correspond well with the phase image.

**Figure 4:**
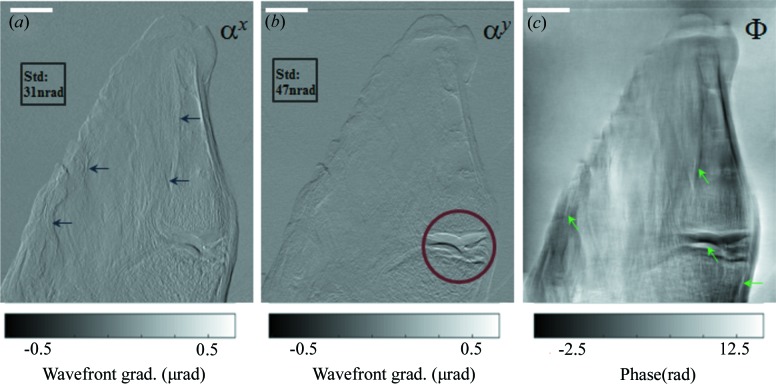
Hard X-ray speckle technique applied to the imaging of a chicken’s wing tip. (*a*) Horizontal and (*b*) vertical wavefront gradients, (*c*) phase contrast obtained with the speckle tracking technique. The scale bar at the top of image is 3.0 mm long.

To demonstrate the proposed method for biological study with higher-energy X-rays, we have carried out experiments on a chicken’s wing tip. Fig. 4 ▸ shows the retrieved horizontal, vertical phase gradient and the reconstructed phase images for the chicken’s wing tip sample by using the proposed Al–Cu alloy. Vertical features (marked by arrows) are clearly visible in the horizontal wavefront gradient image (Fig. 4*a*) ▸ whilst they are hardly noticeable in the vertical gradient (Fig. 4*b*) ▸. As expected, the joint between the phalanges (enclosed by a circle in Fig. 4*b*) ▸ can be clearly seen in Fig. 4(*b*) ▸, but is barely observable in the vertical gradient image (Fig. 4*a*) ▸. As shown in Fig. 4(*c*) ▸, the phase image induced by the sample is reconstructed from the two wavefront gradient images. Both the soft tissues and cartilage (marked by arrows) can be clearly visible in the phase image. The results illustrate that considerable and substantially enhanced information can be gleaned by observing both the phase gradient and phase images at the high-energy region. It shows that the proposed technique can be used to perform phase-contrast imaging to investigate thick biological samples.

## 4. Towards optimization   

To demonstrate the feasibility of other materials as RAMs at high X-ray energies, further experiments were carried out at the B16 test beamline, Diamond Light Source, UK (Sawhney *et al.*, 2010) ▸. A polychromatic beam of X-rays of average energy 55 keV was filtered with 1.5 mm copper plate from the white beam source. The distance between the bending magnet X-ray source and the absorption mask was *L*_1_ = 40 m. The aim was to determine whether improvements could be made to image quality by changing the RAM, which would show that optimization of the technique to specific samples is both possible and worthwhile.

The different combinations of metals in alloys, and the ability to fabricate different textures (*i.e.* grain size), leads to a vast array of possible RAM characteristics that can be made fit for purpose. In addition to the Al–Cu alloy, the Mg–Zn alloy was tested to check the performance of alloys with different element combinations. Here, Mg–Zn alloy was made with a similar process to that for Al–Cu alloy. In addition, we also used two porous materials generally used in the construction of buildings: limestone and mortar. Both the limestone and mortar were cored into a cylinder with a diameter of 4.5 mm. The main chemical component for limestone is carbonate, ∼90% by mass, and the rest is composed of quartz (∼8%) and some clays (∼2%). It has a large open porosity, with a narrow pore size distribution (average pore size is ∼2 µm). The mortar specimen was cast with a 0.51 water-to-cement ratio by mass, 50% volumetric fraction of sand and 19% volumetric fraction of cement. After being cured for three months, it was then dried in an oven at 50°C for 5 d.

To investigate different RAMs, we chose the rhyolite (as this contains a number of phases with which to gauge image quality) and a weakly absorbing, yet structurally complex, biological sample: a beetle enclosed within manmade amber (maximum thickness 20 mm). The samples were placed downstream (*L*_2_ = 0.3 m) of the absorption mask and the sample and detector were set to a maximum achievable distance (*L*_3_ = 0.7 m) to maximize the angular sensitivity. The X-ray camera with an effective pixel size of 3.8 µm × 3.8 µm was based on a pco.edge detector and a macro lens equipped with Ce-doped LuAG scintillator. A stack of 50 images was collected with and without the sample present in the X-ray beam by scanning the RAMs vertically with a step size of μ = 2 µm, and data acquisition for each image was 1.0 s.

**Figure 5:**
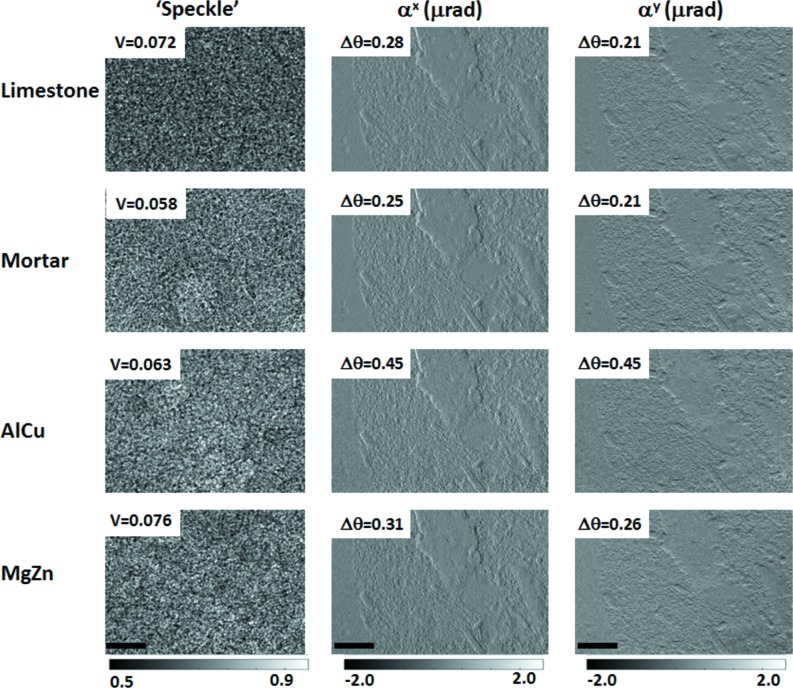
Speckle patterns produced with different RAMs, and the corresponding retrieved horizontal and vertical wavefront gradient images for a rhyolite sample. The scale bar is equal to 2 mm.

The generated ‘speckle’ image with different RAMs and the retrieved wavefront gradient images for the rhyolite are illustrated in Fig. 5 ▸. The limestone returns the most uniform speckle pattern, while the other three are variably patchy. Both limestone and Mg–Zn alloy show slightly higher visibility over 7%, while the ones for the mortar and Al–Cu alloy are about 6%. The Mg–Zn alloy, while not quite as even as the limestone, does return a much finer speckle. Qualitatively, all the retrieved wavefront gradient images seem to be similar to each other. To quantitatively describe the quality for these images (Wang, Kashyap *et al.*, 2015 ▸; Pfeiffer *et al.*, 2007 ▸; Diemoz *et al.*, 2012 ▸), the standard deviation of wavefront gradients in empty space was calculated with 200 × 150 detector pixels. Among these images, the higher angular sensitivity of the wavefront gradients from mortar is achieved with 0.25 µrad and 0.21 µrad for the horizontal and vertical direction, respectively. It turns out that the ones from Al–Cu alloy show the worst angular sensitivity with 0.45 µrad for both directions. Here, we would like to point out that the highest visibility of speckle pattern does not guarantee the best tracking accuracy. The speckle size, transmission of the RAM and speckle visibility will all affect the retrieved phase image quality. Overall, it demonstrates that the image quality can be further improved by choosing appropriate RAMs for the phase-contrast image according to the requirement on the X-ray energy, sample dimensions and spatial resolution.

**Figure 6:**
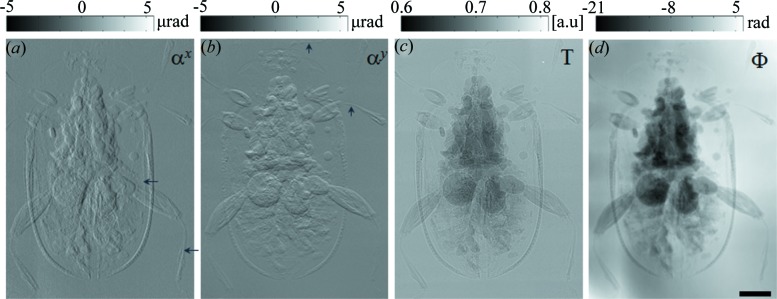
Retrieved differential wavefront gradient, transmission and phase image of manmade amber by using a synchrotron radiation source. (*a*) Horizontal and (*b*) vertical differential wavefront gradient, (*c*) transmission and (*d*) reconstructed phase images of a beetle inside manmade amber. The scale bar is equal to 2 mm.

Finally, Mg–Zn alloy was then used to perform the phase-contrast imaging for a beetle sample to demonstrate the potential application for the study of paleontology with the proposed RAMs. Here, the beetle was embedded in a manmade amber block of dimensions 30 mm (L) × 20 mm (W) × 15 mm (T). Since the sample was larger than the effective field of view (10 mm × 8 mm) of the X-ray camera, 15 stacks of images were collected by two-dimensional raster scanning the sample, and the extracted images were then stitched together (Preibisch *et al.*, 2009 ▸). The retrieved horizontal and vertical differential phase images, transmission and reconstructed phase images are shown in Fig. 6 ▸. As shown in Fig. 6(*c*) ▸, the transmission signal shows edge enhancement from interference due to the high coherence of the X-ray beam. As expected, orthogonal differential phase images present the distinctive image contrast along the horizontal and vertical directions. The beetle tissues can be clearly observed from the retrieved phase images compared with the transmission image. It further confirms that a high-quality X-ray phase-contrast image can be obtained with the proposed RAMs, and the retrieved different contrast images in the higher-energy region can be used to reveal subtle details and provide complementary information.

## 5. Discussion and conclusions   

We have described a new concept to retrieve phase-contrast images by using engineered porous materials as RAMs. We demonstrated that phase images of representative samples can be retrieved with such materials over 50 keV. The results illustrate a significant increase in image contrast compared with conventional absorption-contrast method for both biological and geological samples. We have also shown that the engineering materials are tailorable, and thus can be optimized. The porous building materials were demonstrated to be useful for certain applications. Moreover, the value in using multi-phase alloys is particularly encouraging, with the following advantages. Firstly, by choosing alloy compositions with light and heavy element combination as part of this approach, the speckle-based X-ray imaging technique can cover energy regions typically used in the study of weakly-attenuating materials. Secondly, we note that alloys can be easily modified *via* fabrication processes, which can allow different size and morphology, in particular the porosity, to be specifically designed for the requirement of the RAM, *e.g.* casting methods and subsequently heat treatments. Finally, another valuable feature is that alloys offer the adaptability to achieve either a larger field of view or higher spatial resolution for phase-contrast imaging by simply adjusting the alloys with different pore and grain size. The pore size of the alloys can alternatively be tuned to match the detector pixel size. Based on the above criteria for the RAM, we can choose different engineered porous materials for the phase-contrast imaging according to the working energy and detector pixel size.

Compared with the steel wool we used previously with high-energy X-rays (Wang, Kashyap, Cai *et al.*, 2016 ▸), the engineering materials are easy to use and the experimental setup is comparatively simple. We anticipate that these materials will contribute valuable images at the high-energy X-ray region in a wide range of applications and studies across disciplines.
